# Electroacupuncture Alleviates Osteoarthritis by Suppressing NLRP3 Inflammasome Activation in Guinea Pigs

**DOI:** 10.1155/2020/5476064

**Published:** 2020-09-04

**Authors:** Zhankui Wang, Mo Chen, Bo Wang, Wulin Kang, Hongchao Yu, Xiaoqun Li, Bo Dong, Puwei Yuan

**Affiliations:** ^1^Department of Orthopedics, The First Clinical Medical College, Shaanxi University of Chinese Medicine, Xianyang 712046, Shaanxi, China; ^2^Department of Anesthesiology, Suzhou Municipal Hospital (North District), Nanjing Medical University Affiliated Suzhou Hospital, Suzhou 215008, Jiangsu, China

## Abstract

Osteoarthritis (OA) is an increasingly prevalent disease affecting synovial joints, which includes joint degeneration, inflammation, and joint pain. The activation of nucleotide-binding and oligomerization domain-like receptor containing protein 3 (NLRP3) could promote synovial inflammation. Previous studies have shown that electroacupuncture (EA) has potential anti-inflammatory effect. However, the effect of EA treatment on OA remains unclear. The aim of this study was to investigate the effect of applied EA on OA and joint pain and its relationship with NLRP3 inflammasome. The Hartley guinea pigs with naturally occurring OA at age 18 months were chosen as the OA model and treated with EA for 4 weeks. Mechanical allodynia was quantified by using von Frey filaments. The expression of NLRP3 inflammasome and the downstream proinflammatory factors in the cartilage tissue were quantified. Our results showed that EA treatment significantly reduces mechanical allodynia, improves the articular cartilage structure, and decreases the fibrillation on the cartilage surface in guinea pigs with spontaneous osteoarthritis. Moreover, we also found that EA treatment attenuates the NLRP3 inflammasome activation and suppresses the protein expression levels of caspase-1 and IL-1*β* in the cartilage tissue. Our findings suggest that EA treatment attenuates OA and joint pain by suppressing NLRP3 inflammasome activation and support further investigation of the potential therapeutic tactics.

## 1. Introduction

Osteoarthritis (OA) is the most common joint disease, which is characterized by the degeneration of articular cartilage, synovial inflammation, and subchondral bone rebuilding [1]. OA typically affects the hips, knees, hands, feet, and spine, with a high prevalence of chronic pain. It is estimated that 242 million (3.8% of the population worldwide) people are suffering hip and knee OA [2]. However, there is still no effective pharmacological treatments to slow or reverse the progression of OA. Therefore, it is of crucial importance to explore a potential effective treatment for OA.

Recently, many studies reported that the activation of nucleotide-binding and oligomerization domain-like receptor containing protein 3 (NLRP3) is associated with various arthritic disorders. The activation of NLRP3 inflammasome leads to the production of interleukin-1 beta (IL-1*β*) and tumor necrosis factor alpha (TNF-*α*), which could promote synovial inflammation, cartilage degeneration, and the apoptosis of the chondrocyte [3, 4]. Increasing evidences have highlighted the central role of the NLRP3 inflammasome in the pathogenesis of inflammatory and immune disorders and the pathophysiology of OA [5–7]. These findings indicate that suppressing the activation of NLRP3 inflammasome may be beneficial in the progression of OA.

Electroacupuncture (EA), a traditional Chinese medicine, has attracted increasing attention. It is a form of acupuncture where a small electric current is passed between pairs of acupuncture needles. The anti-inflammatory effect of EA has been reported by a number of studies [8–10]. Our previous study has proved that EA could ameliorate the inflammatory lung injury through inhibition of the high-mobility group box protein 1 (HMGB-1) and IL-1*β* release [11]. However, the effect of applied EA on OA and joint pain and its relationship with NLRP3 inflammasome remain unclear.

In this study, we assessed the effect of EA treatment on OA pain in guinea pigs with spontaneous osteoarthritis. The ability of EA to improve histopathological changes in the OA model and attenuate the NLRP3 inflammasome activation was also investigated. Finally, the expression of proinflammatory factor downstream of NLRP3 and matrix metallopeptidase 13 (MMP13) in the cartilage tissue was assessed.

## 2. Materials and Methods

### 2.1. Animals and Grouping

17-month-old male Hartley guinea pigs, the spontaneous aging-related OA model, and 4-month-old male Hartley guinea pigs were provided by the Experimental Animal Center of Xi Xian New Area (Xi'an, China). The animals were housed at a temperature of 23°C–25°C and a humidity of 45–55%, provided food and water ad libitum, maintained on a 12/12 h light/dark cycle, and adapted to the environment for one week prior to the experiments.

The 17-month-old guinea pigs were randomly divided into the following three groups (*n* = 6 per group): an OA group, an EA + OA group, and a sham EA + OA group. The 4-month-old male Hartley guinea pigs were assigned to the control group. Guinea pigs in the EA + OA group received EA treatment once every other day for 4 weeks. Guinea pigs in the sham EA + OA group received acupuncture needles without electrical stimulation once every other day for 4 weeks.

### 2.2. EA Treatment

EA treatment was performed at “Neixiyan” (Ex-LE4) acupoint, which is located at the medial cavity of the patella and the patellar ligament, and the “Dubi” (ST35) acupoint, located at the lateral cavity of the patella and the patellar ligament. All guinea pigs receiving EA treatment were anaesthetized with intraperitoneal 3% pentobarbital sodium (0.1 ml/100 g). After the guinea pigs were anaesthetized, EA was administered at the 2 Hz frequency for 30 minutes once every other day for 4 weeks by an electronic acupuncture treatment instrument (No. SDZ-IIB; Suzhou Medical Appliances, Suzhou, China), and the intensity was adjusted to induce moderate muscle contraction of the hindlimb. Ex-LE4 and ST35 were chosen based on their effect in reducing osteoarthritis pain [12].

### 2.3. Nociceptive Behavioral Test

The nociceptive behavioral test was performed every 7 days, starting from the first day to 4 weeks. The guinea pigs were first habituated to the testing environment for 30 min. The baseline nociceptive thresholds were tested one day before the experiment. The test was repeated three times, and the mean value was calculated. The mechanical threshold of guinea pigs was measured by using the “up-down” method [13]. After an acclimation period of 30 min, a series of calibrated von Frey filaments (Stoelting, Kiel, WI, USA) were applied perpendicularly to the plantar surface of the left hindpaw with sufficient force to bend the filament for 6 s. Withdrawal or paw flinching was considered as a positive response.

### 2.4. Safranin O/Fast Green and H&E Staining

Changes in the cartilage tissue were examined morphologically as described previously [13]. The cartilage tissue of the guinea pigs was excised, fixed with 4% paraformaldehyde for 24 h, and decalcified in 10% EDTA for 2 weeks. All cartilage tissues were embedded in paraffin before cutting into 5 *μ*m sections and then stained with hematoxylin and eosin (H&E) and safranin O/fast green for microscopic examination. Evaluations were performed by a pathologist blind to experimental groups using an Olympus CH30 microscope.

### 2.5. Western Blot Analysis

Western blot analysis was performed as previously described [14]. The protein concentrations of the cartilage tissue were determined using a BCA Protein Assay Kit (Thermo Fisher Scientific, Inc.). Proteins (30 *μ*g) were separated by 12% SDS-PAGE gel and transferred to a nitrocellulose membrane (Bio-Rad, Hercules, California, USA). The nitrocellulose membrane was blocked with 5% nonfat dry milk for 2 h at room temperature, followed by an incubation with a primary antibody against NLRP3 (1 : 500; cat. no. ab214185; Abcam, Cambridge, MA, USA), caspase-1 (1 : 500; cat. no. ab1872; Abcam, Cambridge, MA, USA), IL-1*β* (1 : 500; cat. no. ab150777; Abcam, Cambridge, MA, USA), and MMP13 (1 : 3000; cat. no. ab39012; Abcam, Cambridge, MA, USA) at 4°C overnight and then an incubation with a secondary antibody (goat anti-rabbit IgG; 1 : 10,000; Cat. No. ab6721; Abcam) conjugated with horseradish peroxidase for 2 h at room temperature. The proteins were detected using Pierce ECL Plus Western Blotting Substrate (cat. no. 32134; Thermo Fisher Scientific, Inc.). *β*-Actin levels (Sigma-Aldrich, Merck KGaA, Darmstadt, Germany; 1 : 500) were used as the control. A densitometry analysis was performed using ImageJ software (version 1.8.0; National Institutes of Health, Bethesda, MD, USA).

### 2.6. ELISA for the TNF-*α* and IL-1*β* Concentrations in Serum

The protein content was measured by a BCA Protein Assay Kit (Thermo Fisher Scientific, Inc.). The levels of TNF- *α* and IL-1*β* in serum were measured using ELISA kits (R&D Systems, Inc.) following the manufacturer's protocol.

### 2.7. Data Analysis and Statistics

The data were expressed as mean ± standard deviation. Analysis was performed using GraphPad Prism v7.00 software (GraphPad Software, California, USA). One-way analysis of variance was conducted for multiple comparisons. *P* value < 0.05 was considered statistically significant.

## 3. Results

### 3.1. EA Reduces Mechanical Allodynia in Guinea Pigs with Spontaneous Osteoarthritis

**Figure 1:**
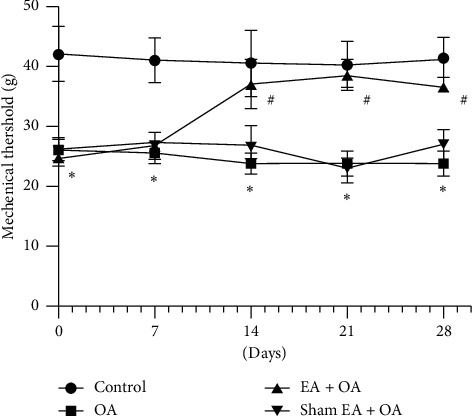
Time course of the effects of EA on OA-induced mechanical allodynia. Time course of mechanical withdrawal threshold in response to von Frey filaments in the four groups (*n* = 6, ^*∗*^*P* < 0.05, OA group vs. control group. ^#^*P* < 0.05, EA + OA group vs. OA group).

As shown in Figure 1, 18-month-old guinea pigs with spontaneous osteoarthritis in the OA group had a significantly reduced mechanical withdraw threshold when compared with the control group (^*∗*^*P* < 0.05, *n* = 6). Compared with the OA group, EA treatment significantly increased the mechanical threshold from day 14 (^#^*P* < 0.05, *n* = 6). However, sham EA had no significant effect on the paw withdraw threshold.

### 3.2. EA Improves Histopathological Changes in OA Models

**Figure 2:**
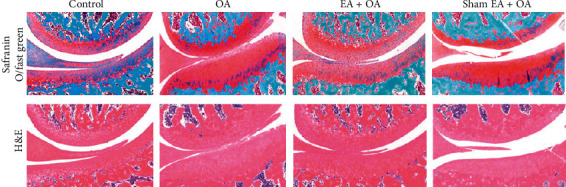
Representative images of tibial cartilage of the four groups stained with safranin O (up) and H&E (down) (magnification ×200).

In 18-month-old guinea pigs of the OA group, the cartilage surface was not smooth, and fibrillation was observed when compared with the control group in safranin O staining (Figure 2, up) and H&E staining (Figure 2, down). After EA treatment, the articular cartilage structure was improved, and the fibrillation on the cartilage surface was decreased when compared with the OA group. No significant effect was observed in the sham EA + OA group.

EA treatment attenuates the NLRP3 inflammasome activation and suppresses the proinflammatory factor downstream of NLRP3 in the cartilage tissue.

**Figure 3:**
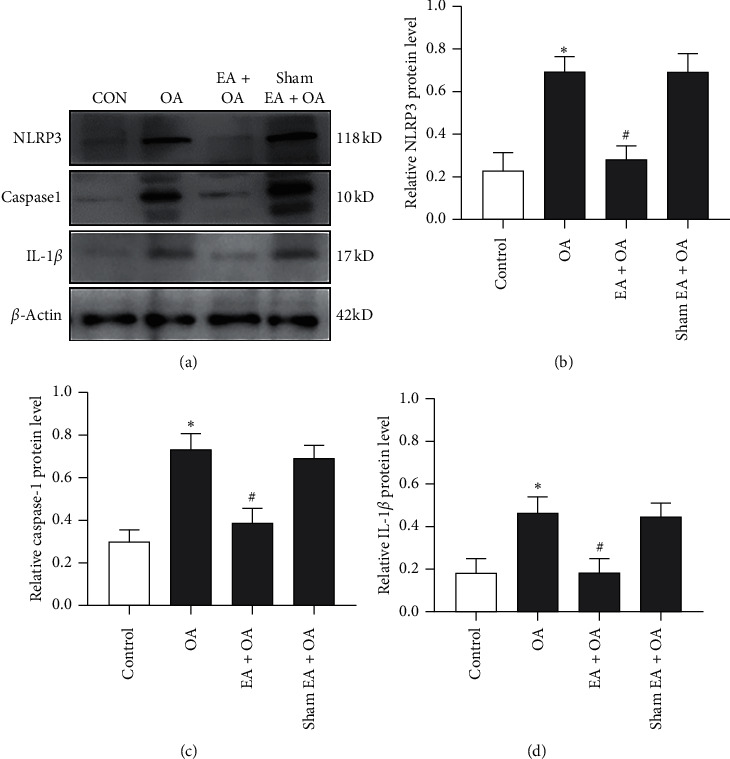
Expression of NLRP3, caspase-1, and IL-1*β* in the cartilage tissue of the four groups. (a) Representative results of Western blot for NLRP3, caspase-1, and IL-1*β* expression in the cartilage tissue of the four groups. (b–d) Immunoblot analysis of NLRP3, caspase-1, and IL-1*β* in the cartilage tissue of each group (*n* = 6, ^*∗*^*P* < 0.05 OA group vs. control group. ^#^*P* < 0.05 EA + OA group vs. OA group).

As presented in Figure 3(b), the protein expression of NLRP3 in the cartilage tissue increased significantly in the OA group (^*∗*^*P* < 0.05, *n* = 6) when compared with the control group. Compared with the OA group, the guinea pigs that received EA treatment showed a significant decrease in the expression of NLRP3 (^#^*P* < 0.05, *n* = 6). However, there was no significant difference in NLRP3 expression in the cartilage tissue between the OA group and sham EA + OA group. To detect the activation of the NLRP3 downstream pathway, we also evaluate the protein expression levels of caspase-1 and IL-1*β* in the cartilage tissue. As shown in Figures 3(c) and 3(d), the expression trends of caspase-1 and IL-1*β* were similar as NLRP3 in the four groups. These findings showed that EA treatment may suppress the activation of the NLRP3 inflammasome and downstream pathway.

### 3.3. EA Suppresses OA-Induced TNF-*α* and IL-1*β* Production

**Figure 4:**
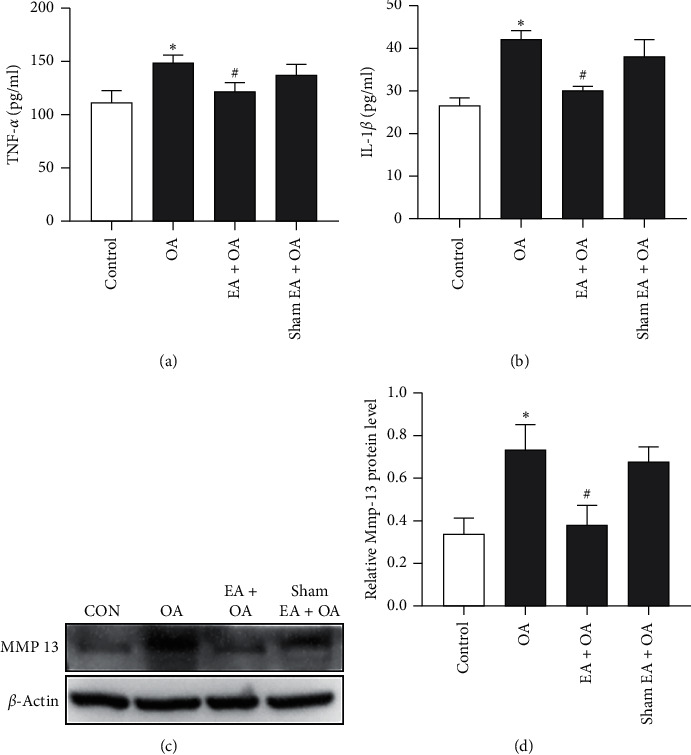
TNF-*α* and IL-1*β* concentrations in serum and protein expression of MMP13 in the cartilage tissue of the four groups. (a, b) In serum, both TNF-*α* and IL-1*β* concentrations increased significantly in the OA group when compared with the control group (^*∗*^*P* < 0.05, *n* = 6). EA treatment remarkably decreased the TNF-*α* and IL-1*β* production in the EA + OA group (*n* = 6, ^#^*P* < 0.05 vs. OA group). (c, d) The effect of EA on cartilage matrix metabolism. The expression of MMP13 increased significantly in the OA group when compared with the control group (^*∗*^*P* < 0.05, *n* = 6). EA treatment remarkably decreased the expression of MMP13 of the cartilage in the EA + OA group (*n* = 6, ^#^*P* < 0.05 vs. OA group).

Considering that TNF-*α* and IL-1*β* are important mediators of inflammatory response, we also examined the levels of TNF-*α* and IL-1*β* in the serum. As indicated in Figures 4(a) and 4(b), the concentrations of TNF-*α* and IL-1*β* in the serum were significantly increased in the OA group compared with the control group (^*∗*^*P* < 0.05, *n* = 6). In contrast to the OA group, EA remarkably decreased the TNF-*α* and IL-1*β* production in the EA + OA group (^#^*P* < 0.05, *n* = 6). However, there were no significant differences in the TNF-*α* and IL-1*β* concentrations in the serum between the OA group and sham EA + OA group. The results suggested that EA could suppress OA-induced TNF-*α* and IL-1*β* production in the serum.

To assess the effect of EA on cartilage matrix metabolism, we also analyzed the expression of MMP13 in the cartilage tissue. As shown in Figures 4(c) and 4(d), compared with the control group, the expression of MMP13 in the articular cartilage tissue of the OA group was significantly enhanced (^*∗*^*P* < 0.05, *n* = 6). Compared with the OA group, the guinea pigs that received EA treatment showed a significant decrease in the expression of MMP13 (^#^*P* < 0.05, *n* = 6). However, there was no significant difference in MMP13 expression in the cartilage tissue between the OA group and sham EA + OA group. These results revealed that EA may play an important role in the regulation of cartilage matrix metabolism.

### 3.4. Effect of EA Treatment Can Last for Some Time When Stopping It

**Figure 5:**
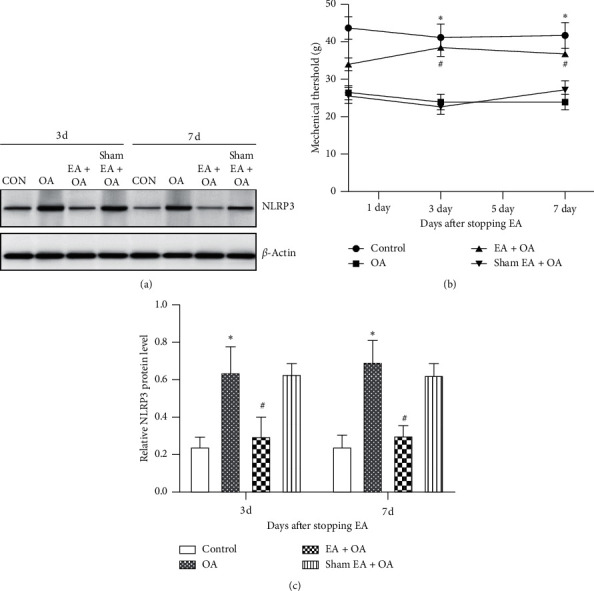
Expression of NLRP3 in the cartilage tissue and changes of mechanical withdrawal threshold of the four groups when stopping EA treatment. (a) Representative results of Western blot for NLRP3 expression in the cartilage tissue of the four groups. (b) Immunoblot analysis of NLRP3 in the cartilage tissue of each group (*n* = 6, ^*∗*^*P* < 0.05 OA group vs. control group. ^#^*P* < 0.05 EA + OA group vs. OA group). (c) Time course of mechanical withdrawal threshold in response to von Frey filaments in the four groups (*n* = 6, ^*∗*^*P* < 0.05, OA group vs. control group. ^#^*P* < 0.05, EA + OA group vs. OA group).

In order to see how long that EA may last for alleviation of OA, the behavioral test and the expression of NLRP3 inflammasome were measured at 3 days and 7 days after stopping EA treatment. And our results suggested that the expression level of NLRP3 inflammasome still showed a significant decrease in the EA + OA group on days 3 and 7 after stopping EA, compared to the OA group (Figures 5(a) and 5(b)). The expression trend of NLRP3 inflammasome was similar as that in the group of EA treatment (^*∗*^*P* < 0.05, *n* = 6). Moreover, the mechanical withdraw threshold demonstrated that the analgesic effect of EA can also last at least 7 days even after stopping EA treatment (Figure 5(c)). These findings showed that the effect of EA treatment may last at least 7 days after stopping it.

### 3.5. “Neixiyan” and “ST35” Acupoints Might Be Specific for OA Therapy

**Figure 6:**
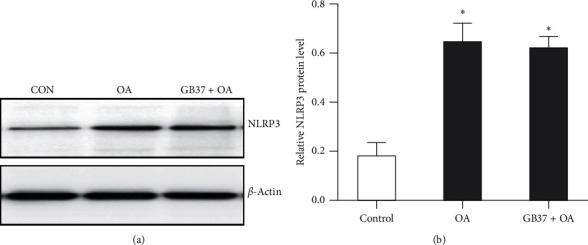
Expression of NLRP3 in the cartilage tissue when acupuncturing at GB37. (a) Representative results of Western blot for NLRP3 expression in the cartilage tissue of the four groups. (b) Immunoblot analysis of NLRP3 in the cartilage tissue of each group (*n* = 6, ^*∗*^*P* < 0.05 vs. control group).

To determine whether “Neixiyan” and “ST35” acupoint is specific for OA therapy, an irrelevant acupoint (GB37) was chosen as a control. And our results showed that the expression level of NLRP3 inflammasome in the GB37 + OA group was not changed significantly compared to the sham group by Western blot analysis (Figure 6). Therefore, we concluded that acupuncture at GB37 cannot directly decrease the expression of NLRP3 inflammasome. To sum up, we believe that “Neixiyan” and “ST35” acupoint might be specific for OA therapy.

## 4. Discussion

OA is an increasingly prevalent disease affecting synovial joints. The most significant symptom is debilitating chronic OA pain, and pharmacological treatments do not provide effective analgesia [15]. The pathogenesis involves various factors such as mechanics, genetics, and aging [16]. The aim of this study was to explore the potential therapeutic effect of EA on OA and chronic joint pain and its relationship with NLRP3 inflammasome. The Hartley guinea pig spontaneously develops OA similar to that of human OA in the knee joint; thus, it is used as an aging-related OA model. According to a previous study, we chose Hartley guinea pigs with naturally occurring OA at age 18 months as the OA model, and the Hartley guinea pigs at age 5 months were selected as control [17].

In this study, we demonstrated that EA treatment significantly reduces mechanical allodynia, improves the articular cartilage structure, and decreases the fibrillation on the cartilage surface in guinea pigs with spontaneous osteoarthritis. Moreover, we also found that EA treatment attenuates the NLRP3 inflammasome activation and suppresses the protein expression levels of caspase-1 and IL-1*β* in the cartilage tissue, and that sham EA had no effect. Our study provided the evidence that EA could alleviate osteoarthritis and chronic OA pain by suppressing NLRP3 inflammasome activation in guinea pigs with spontaneous osteoarthritis.

NLRP3 inflammasome activation could lead to caspase-1 activation, which causes the maturation of IL-1*β* [18, 19]. Caspase-1 is known as an inflammatory caspase that plays a crucial role in the maturation of pro-IL-1*β* into active cytokine [20]. IL-1*β* is a potent proinflammatory mediator in many immune reactions, including the recruitment of innate immune cells to infected sites and modulation of adaptive immune cells [21]. Increasing evidences support the conclusion that the aberrant activation of the NLRP3 inflammasome is associated with autoinflammatory and chronic inflammatory diseases [22, 23]. Cheng et al. demonstrated that dexmedetomidine could inhibit the NF-*κ*B pathway and NLRP3 inflammasome to reduce the maturation and release of inflammatory factors in the cartilage tissue to attenuate papain-induced OA in rats [24].

EA is a form of acupuncture where a small electric current is passed between pairs of acupuncture needles and has been known to play a crucial role in inducing anti-inflammatory responses [8–10, 25]. Chronic pain is often persistent and poorly treated, which is an important focus in research of mechanism of acupuncture analgesia. It has been proved that EA is effective in relieving chronic pain in patients with knee osteoarthritis [26]. However, the involved mechanisms remain unclear. In our previous study, we found that EA pretreatment reduces inflammatory lung injury by suppressing the activation of the NLRP3 inflammasome, decreasing caspase-1 and IL-1*β* levels, and reducing the inflammatory response [27]. However, the effect of applied EA on NLRP3 inflammasome in OA remains unclear. In this study, we found that OA increased the expression of NLRP3, caspase-1, and IL-1*β* in the cartilage tissue. EA could attenuate histopathological damages in OA. We also detected the protein expression of NLRP3 and downstream proinflammatory factors to confirm the suppressive effect of EA treatment on NLRP3 inflammasome activation. However, there are some limitations in this study. The suppressive effect of EA treatment on OA-induced NLRP3 inflammasome activation might be just one aspect of the protective effect of EA. Other directly or indirectly molecules and pathways could also be regulated by EA treatment. The exact mechanism of the inhibition of NLRP3 inflammasome activation mediated by EA treatment needs further research. Additionally, the efficacy of EA treatment in specific immune cell types should also be investigated.

## 5. Conclusions

In conclusion, EA treatment could reduce mechanical allodynia, attenuate the NLRP3 inflammasome activation, and suppress the proinflammatory factor downstream of NLRP3. Our findings demonstrate that EA may alleviate osteoarthritis by suppressing NLRP3 inflammasome activation in guinea pigs with spontaneous osteoarthritis.

## Data Availability

All data generated or analyzed during this study are included in this published article.
